# Phenol hemihydrate: redetermination of the crystal structure by neutron powder diffraction, Hirshfeld surface analysis and characterization of the thermal expansion

**DOI:** 10.1107/S2056989020007719

**Published:** 2020-06-12

**Authors:** A. Dominic Fortes

**Affiliations:** a ISIS Neutron & Muon Spallation Facility, Rutherford Appleton Laboratory, Harwell Science and Innovation Campus, Chilton, Oxfordshire, OX11 0QX, England

**Keywords:** phenol, hydrate, neutron powder diffraction, thermal expansion, DFT, Hirshfeld surface analysis

## Abstract

Phenol hemihydrate, C_5_H_5_OH·0.5H_2_O, crystallizes in the space group *Pbcn*, *Z* = 8. The previously published crystal structure [CSD refcode PHOLHH; Meuthen & von Stackelberg (1960 ▸). *Z. Elektrochem*. **64**, 387–390] is shown to be incorrect. Pairs of phenol mol­ecules, related by an inversion centre, are bridged by one water mol­ecule *via* O—H⋯O hydrogen bonds; an extended *R* ^4^_4_(8) hydrogen-bonded motif links these inversion dimers into chains parallel to the *c* axis. Packing of the chains is achieved by weaker T-shaped C—H⋯π inter­actions between nearest neighbour phenol mol­ecules in the *bc* plane. Analysis of the thermal expansion and parameterization with a Debye model in terms of the linear elastic moduli shows that the *c* axis is ∼3 times stiffer than the two orthogonal directions.

## Abstract 2

The existing crystal structure of phenol hemihydrate is shown to be incorrect and a new model, refined from high-resolution neutron powder diffraction data, is reported.

## Chemical context   

Phenol is the simplest aromatic alcohol; as such, it is one of the most straightforward systems in which to study the competition between medium-strength O—H⋯O hydrogen bonds, π⋯π/C—H⋯π inter­actions and steric effects on packing in the solid state (Zavodnik *et al.*, 1988 ▸: Allan *et al.*, 2002 ▸). However, there are sparse structural data on compounds formed solely between phenol and simple polar mol­ecules such as water or ammonia. The binary phenol–water system is characterized by a wide region of liquid immiscibility (Smith, 1932 ▸); liquids on the phenol-rich side exhibit a propensity to supercool and crystallize solid phenol rather than an inter­mediate hydrated compound. Consequently, early reports of a hemihydrate by Calvert (1865 ▸) proved difficult to reproduce (Alexeev, 1883 ▸: Paternò & Ampola, 1897 ▸). Rapid freezing of stoichiometric liquids with dry ice or liquid air was found to reproducibly form seed crystals of the hemihydrate for structural characterization (Smits & Maarse, 1911 ▸: Rhodes & Markley, 1921 ▸). Although the hydrate melts quite close to room temperature (*T*_m_ = 289 K), the non-H-atom crystal structure of phenol hemihydrate was not reported for several more decades (Meuthen & von Stackelberg, 1960 ▸; CSD refcode PHOLHH).

**Figure 1:**
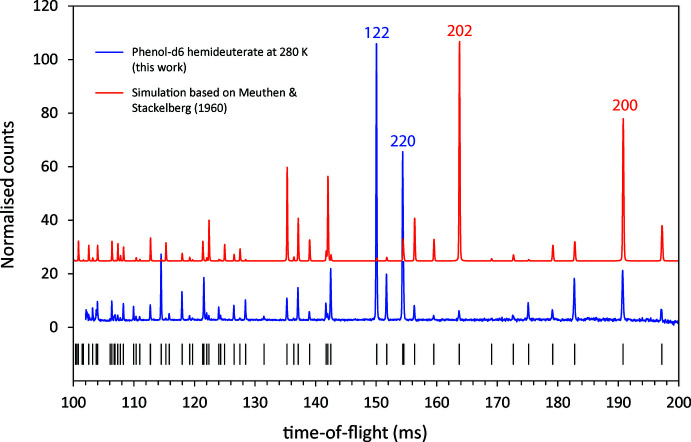
Time-of-flight (TOF) data collected in HRPD’s backscattering detectors over the range 100–200 ms at 280 K (blue) compared with a simulated diffraction pattern (red) based on the structure model of Meuthen & von Stackelberg (1960 ▸). The latter structure was ‘completed’ with geom­etrically positioned deuterons 1.080 Å from, and co-planar with, the phenyl carbons, a deuteron placed 0.990 Å from the hydroxyl oxygen along the O–O vector that gave the smallest C—C—O—D torsion angle; the symmetry-unique water deuteron was placed 0.990 Å from the water oxygen along the remaining O–O vector. In the experimentally observed diffraction pattern, the strongest Bragg peaks in this TOF range are 122 and 220. In contrast, the intensity of 122 from the simulated pattern is extremely weak and 202 has the greatest intensity instead.

As part of a wider study into the structures of complexes formed between simple alcohols and water or ammonia (Fortes, 2019 ▸), neutron powder-diffraction data were collected from a perdeuterated analogue of phenol hemihydrate with the simple objective of determining the hydrogen-atom positions. However, the intensities of the observed Bragg peaks differed so greatly from the calculated intensities as to be irreconcilable with a structure that was generally correct but merely incomplete (Fig. 1 ▸). In conclusion, Meuthen & von Stackelberg’s structure model is incorrect; the neutron powder data were used to determine the correct structure, as reported below.

## Structural commentary   

**Figure 2:**
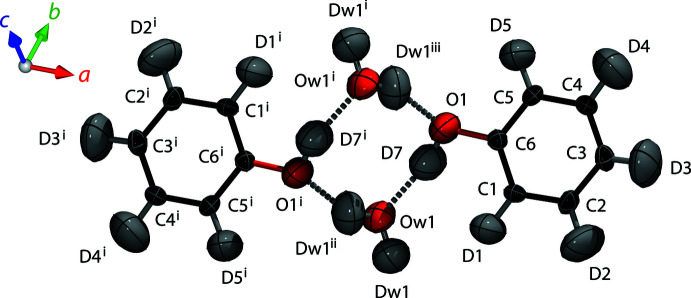
Phenol hemihydrate’s asymmetric unit (right) and the inversion-related atoms required to form the dimer (left); the inversion centre is located in the middle of the ring. Displacement ellipsoids are drawn at the 50% probability level. [Symmetry codes: (i) −*x*, 1 − *y*, 1 − *z*; (ii) −*x*, *y*, 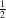 − *z*; (iii) *x*, 1 − *y*, 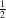 + *z*]

**Figure 3:**
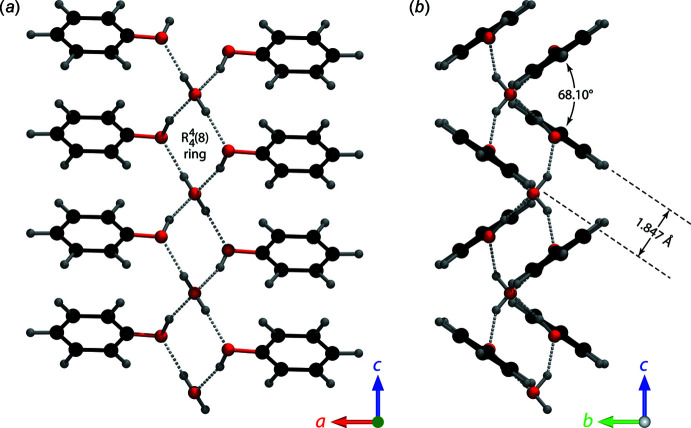
(*a*) The dimer shown in Fig. 2 ▸ extends as a chain parallel to the *c* axis. (*b*) The chain viewed parallel to the *c* axis reveals the offset of the phenyl rings in each dimer and the hinge angle between successive dimers along the chain.

Phenol hemihydrate, (C_5_H_5_OH)·0.5H_2_O, forms an inversion dimer, such that there is only one symmetry-independent phenol mol­ecule per formula unit with atoms on general positions 8 *d*; the bridging water mol­ecule occupies the higher symmetry 4 *c* site associated with a twofold rotation axis (Fig. 2 ▸). The phenyl rings are flat, atoms C1–C6 lying no more than 0.005 Å from a least-squares plane (LSP) fitted through the carbon atoms; hydrogen atoms D1–D5 and the hydroxyl oxygen, O1, lie within 0.041 Å of the same LSP. The hydroxyl deuteron, D7, lies 0.192 Å out of the LSP, leading to a dihedral angle C1—C6—O1—D7 of 17.1 (6)°. The hydroxyl moiety is in a trigonal coordination, both donating and accepting one hydrogen bond from neighbouring water mol­ecules. These hydrogen bonds generate a rhombic motif involving two water mol­ecules and two hydroxyl moieties (Fig. 2 ▸), which may be described by the graph-set notation 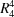
(8). The water mol­ecules are in a tetra­hedral coordination, connecting the 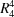
(8) rings to form an infinite chain of inversion dimers extending parallel to the *c* axis (Fig. 3 ▸ *a*). Inversion-related phenol pairs sit on planes with a vertical separation of 1.847 Å and these are in turn canted alternately along the *c* axis so as to form sheets that are co-planar with (011) and (0
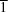
1) (inter­planar angle = 71.92°). Individual phenyl rings are tilted slightly with respect to these sheets such that the hinge angle between directly adjacent pairs of phenol LSPs is reduced to 68.10° (Fig. 3 ▸ *b*).

More importantly, the C—O bonds of the two symmetry-related phenol mol­ecules involved in the dimer are approximately co-aligned with the *a* axis of the crystal, and this marks the principal point of difference with the structure model reported by Meuthen & von Stackelberg (1960 ▸). They correctly inferred the coordination environment of the O atoms and used trial-and-error methods [complemented by a Fourier map projected on (001)] to determine the arrangement of the phenyl rings. Hence, their model contains the same chains of corner-linked four-sided rings extending parallel to the *c* axis, but it differs from the correct structure by having the inversion dimers aligned approximately along the body diagonals of the unit cell. This leads to a significantly different packing of the phenol mol­ecules, as outlined below.

## Supra­molecular features   

**Figure 4:**
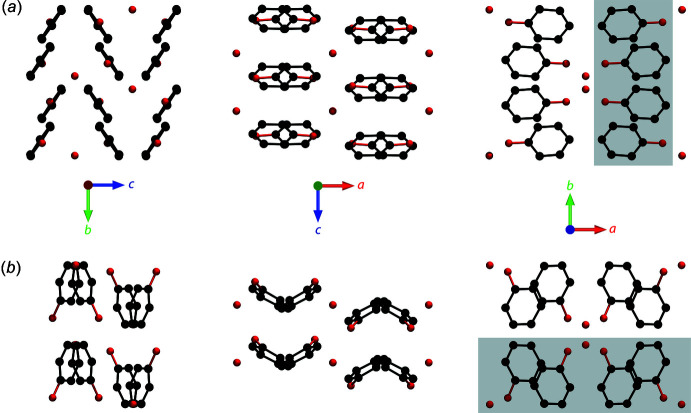
Mol­ecular packing in phenol hemihydrate (*a*) from this work and (*b*) from Meuthen & von Stackelberg (1960 ▸). Hydrogen atoms are omitted for clarity. Shaded rectangles indicate sections through slabs of closest-packed phenol mol­ecules, which are drawn explicitly in Fig. 5 ▸.

Inter­molecular hydrogen-bond lengths and angles are reported in Table 1 ▸. Fig. 4 ▸ compares the unit-cell contents, viewed along each crystallographic axis, between the structure published by Meuthen & von Stackelberg (1960 ▸) and this work. As noted above, Meuthen & von Stackelberg constructed a Fourier map projected on (001) and, clearly, the mol­ecular structure viewed along *c* is quite similar to that obtained here. However, the orientation of the phenol mol­ecules out of the (001) plane differs such that the (100) and (010) projections are completely different.

**Figure 5:**
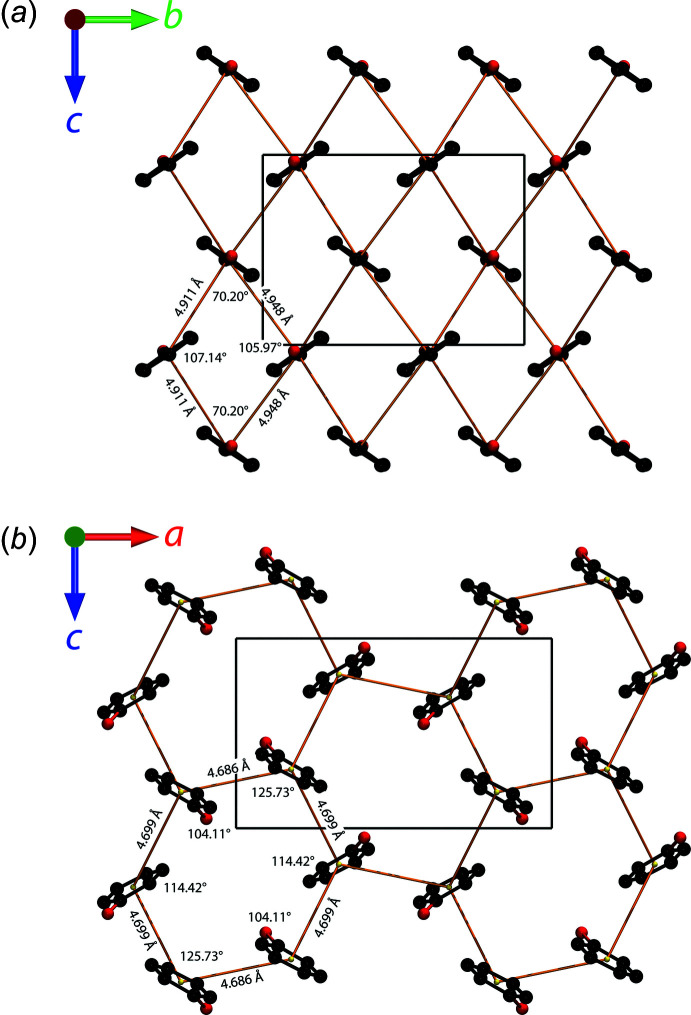
Illustration of the nearest-neighbour inter­actions in the slabs of inter­locking phenol mol­ecules (*a*) from this work and (*b*) from Meuthen & von Stackelberg (1960 ▸). Hydrogen atoms are omitted for clarity and the network of mol­ecular centres is indicated by solid yellow rods.

Phenol mol­ecules from one of the chains shown in Fig. 3 ▸ *a* inter­lock with those of an adjacent chain. The resultant slab of nearest-neighbour phenol mol­ecules lies in the *bc* plane, a cross-section of which is represented by the grey rectangle in Fig. 4 ▸ *a*. An isolated view of the slab along the *a* axis (Fig. 5 ▸ *a*) reveals a rhombic array of T-shaped C—H⋯π inter­actions with a mean separation of 4.93 Å between mol­ecular centres. The equivalent slab of nearest-neighbour phenol mol­ecules in Meuthen & von Stackelberg’s structure model lies in the *ac* plane (grey rectangle in Fig. 4 ▸ *b*); when viewed along the *b* axis (Fig. 5 ▸ *b*), a skewed hexa­gonal array of mol­ecular centres is found in which the inter­actions involve both T-shaped C—H⋯π contacts and offset π–π stacking. The mean distance between mol­ecular centres is 4.69 Å.

## Hirshfeld surface analysis   

**Figure 6:**
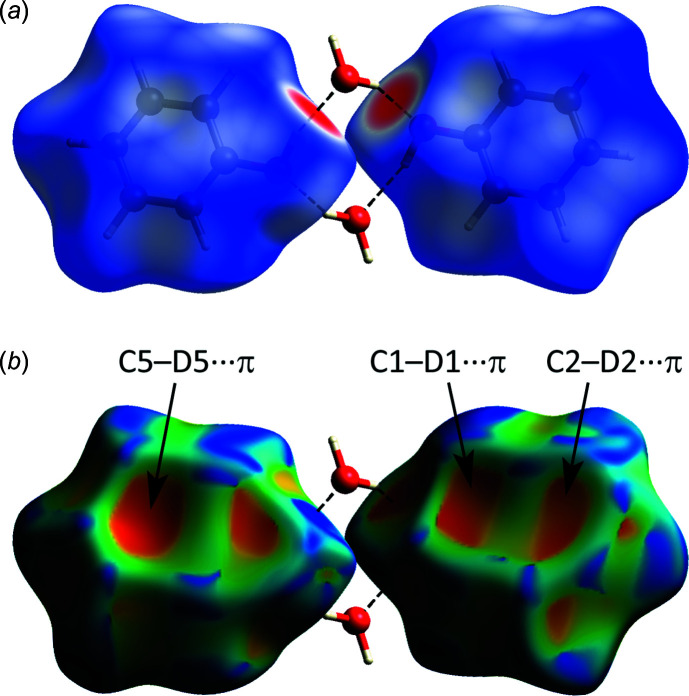
Hirshfeld surfaces of phenol hemihydrates, superimposed on the inversion dimer shown in Fig. 2 ▸. Surfaces are shaded by (*a*) *d_norm_* value and (*b*) the shape-index. Regions of important inter­molecular contacts referred to in the text are labelled (see also Table 1 ▸).

A useful method of analysing and comparing inter- and intra­molecular inter­actions is by calculation of a Hirshfeld surface (Spackman & Jayatilaka, 2009 ▸) and derivation of two-dimensional fingerprint plots (McKinnon *et al.*, 2007 ▸). These have been computed for the structure obtained in this work, for Meuthen & von Stackelberg’s structure model (PHOLHH) and for solid phenol (PHENOL03; Zavodnik *et al.*, 1988 ▸) using *CrystalExplorer 17.5* (Turner *et al.*, 2017 ▸). The *d*_norm_ plot for the correct structure of phenol hemihydrate (Fig. 6 ▸ *a*) was found *via* calculation of the external (*d*_e_) and inter­nal (*d*_i_) distances between pairs of nuclei with a scaled colour of −0.6026 a.u. (red) to 1.1002 a.u. (blue). A corresponding plot of the shape-index was generated in the range of −1.0 to 1.0 a.u. (Fig. 6 ▸ *b*).

Red areas on the Hirshfeld surface indicate contacts that are shorter than the sum of the van der Waals radii and blue areas show where the contacts are longer than the vdW sum. Clearly, there are red patches on the Hirshfeld surface that correspond only with the O—H⋯O hydrogen bonds. For Meuthen & von Stackelberg’s structure model (see Fig. S1 in the supporting information) there are additional red patches in proximity to some of the ring hydrogen atoms, indicating some very short C—H⋯O, and even H⋯H, inter­actions. Weaker C—H⋯π inter­actions are more clearly elucidated from the shape-index plot (Fig. 6 ▸ *b*), where the strongly negative (red) regions delineate specific labelled contacts.

**Figure 7:**
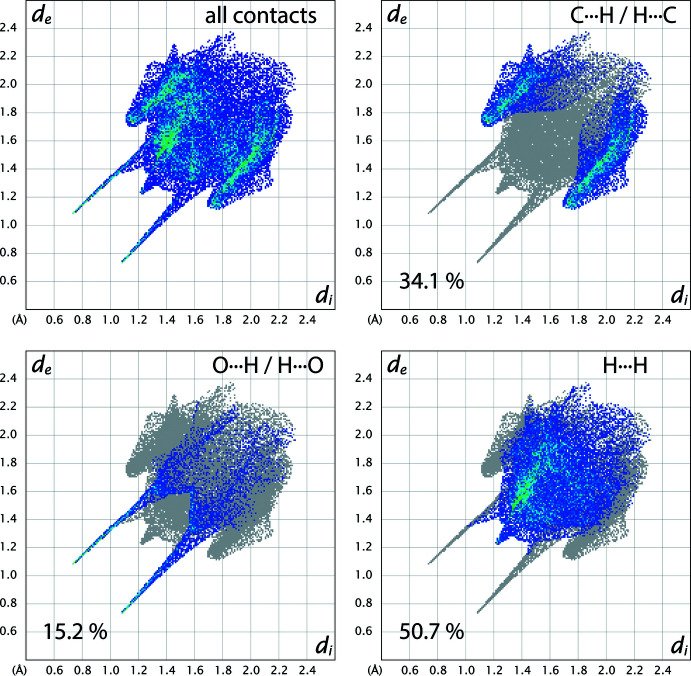
Two-dimensional fingerprint plots showing the distribution of inter­atomic contacts on the Hirshfeld surface of the phenol mol­ecule in phenol hemihydrate.

Two-dimensional fingerprint plots are shown in Fig. 7 ▸. The O⋯H/H⋯O hydrogen-bonded contacts appear as the two sharply pointed regions spreading to the top right from *d*_i_ + *d*_e_ ≃ 1.82 Å; these represent 15.2% of the Hirshfeld surface around the phenol mol­ecule. The next largest contribution to the surface area (34.1%) comes from C⋯H/H⋯C inter­actions, which appear on the fingerprint plots as two approximately symmetrical round-tipped ‘wings’ spreading to the top right from *d*_i_ + *d*_e_ ≃ 2.82 Å, and these represent the T-shaped C—H⋯π contacts. The balance of the Hirshfeld surface area (50.7%) comes exclusively from H⋯H contacts. There are no contributions from C⋯C, C⋯O or O⋯O contacts.

The fingerprint plots for solid phenol (supplementary Fig. S2) are strikingly similar to those for phenol hemihydrate. There are three symmetry-inequivalent phenol mol­ecules in the asymmetric unit of phenol, and the average surface area contributions for the various contacts are: O⋯H/H⋯O = 15.9%; C⋯H/H⋯C = 33.1%; H⋯H = 49.9%; C⋯C = 0.5%; C⋯O/O⋯C = 0.6%. Despite the substantial influence of O—H⋯O hydrogen bonds on the packing, the principal inter­action between phenyl rings in solid phenol is still *via* T-shaped C—H⋯π contacts.

By contrast, the fingerprint plots for Meuthen and von Stackelberg’s phenol hemihydrate structure model (supplementary Fig. S3) display strong indicators that their solution is not correct. In particular, there are some very short H⋯H contacts, with *d*_i_ + *d*_e_ ≃ 1.39 Å, and these comprise 55.3% of the surface area. As it is a matter of trivial geometry to calculate on paper the positions of the ring hydrogens and then to compute inter­atomic distances, it should have been obvious from the outset that their structure was incorrect. The remainder of the contributions to the Hirshfeld surface area are as follows: O⋯H/H⋯O = 14.3%; C⋯H/H⋯C = 26.6%; C⋯C = 3.3%; C⋯O/O⋯C = 0.5%. Note that the significant C⋯C contribution is due to the offset π–π stacking that is apparent from Fig. 5 ▸ *a*.

## Thermal expansion   

Lattice parameters of phenol hemihydrate determined between 10 and 280 K are reported in supplementary Table S1 and plotted in supplementary Fig. S4. Precision at low temperatures is significantly poorer due to substantial strain broadening of the Bragg peaks. These data have been fitted with a second-order Grüneisen approximation to the zero-pressure equation of state [Equation (1)]. In this approximation, the thermal expansion is considered equivalent to elastic strain such that,
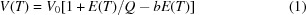
where *V*_0_ is the unit-cell volume at zero pressure, *b* = 1/2(*K*_0_′ − 1) and *Q* = (*V*_0_ *K*_0_/γ); *K*_0_ is the zero pressure isothermal bulk modulus, *K*_0_′ is its first derivative with respect to pressure, and γ is the thermal Grüneisen parameter. The inter­nal energy due to lattice vibrations, *E*(*T*), is then determined *via* a Debye model: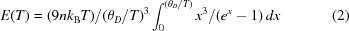 where θ_D_ is the Debye temperature, *n* is the number of atoms per formula unit, and *k*_B_ is the Boltzmann constant; the integral term is evaluated numerically. In order to be dimensionally correct, the individual lattice parameters were fitted as *a* ^3^, *b* ^3^ and *c* ^3^; the fit parameters (along with a fit to the unit-cell volume) are given in supplementary Table S2. The values of *K*_0_/γ reported for each axis therefore correspond with, *e.g*., *K_a_*/γ = –*a* ^3^ (d*P*/d*a* ^3^).

Supplementary Fig. S5 shows the linear and volume thermal expansion coefficients as a function of temperature. The thermal expansivity of the *a* axis (α_1_) differs substanti­ally from that of *b* or *c* (α_2_ and α_3_, respectively), reflected in their very different Debye temperatures and their derived elastic moduli. The linear incompressibility of the *c* axis (assuming no anisotropy of γ) is almost three times larger than the two orthogonal directions, although this remains to be confirmed by any high-pressure studies. The observed behaviour along *a* and *b* is due to weaker dispersion inter­actions between and within the nearest-neighbour slabs (Fig. 5 ▸ *a*) whereas the behaviour along *c* is governed by O—H⋯O hydrogen bonds in the chains of corner-linked 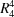
(8) rings.

## Database survey   

Searches of the Cambridge Structural Database (CSD Version 5.41, March 2020 update; Groom *et al.*, 2016 ▸) were carried out to identify structures with geometrically similar O⋯O hydrogen-bonding motifs and similar distorted T-shaped C—H⋯π motifs.

Square rings comprised of two alcohol O–H groups and two water mol­ecules are comparatively uncommon in organic crystals; examples include CSD refcodes KONTIQ (Demirtaş *et al.*, 2011 ▸), AYOPIO (Chantrapromma *et al.*, 2011 ▸), CERYIK (Zhang *et al.*, 2018 ▸) and VABKOA (Li *et al.*, 2010 ▸). However, the compound most closely related structurally to phenol hemihydrate that contains this motif is TMBUOL (2,3,3-trimethyl butan-2-ol hemihydrate; Pachler & von Stackelberg, 1963 ▸).

The rhombic motif of C—H⋯π inter­actions generated over 2800 hits in the CSD search, of which the most inter­esting are the closely related mono-substituted benzenes: chloro­benzene (MCBENZ; Biswas, 1958 ▸; André *et al.*, 1971 ▸; Nath & Naumov, 2015 ▸), bromo­benzene (ZZZSPA; Biswas, 1958 ▸) and iodo­benzene (REKYAI; Merz, 2006 ▸). Each of these crystallizes in space-group type *Pbcn* and adopts a near identical mol­ecular packing of the phenyl rings to that observed in phenol hemihydrate. The lattice parameters of C_6_H_6_Cl are very similar to (C_6_H_5_OH)·0.5H_2_O and the principal difference on substitution of Br and I is an increase in the length of the *a* axis as the length of the carbon–halogen bond increases, these being roughly co-aligned with *a* in the same fashion as the C—O bond in phenol hemihydrate. Similarly, both thio­phenol (JUJPEL; Thomas *et al.*, 2015 ▸) and seleno­phenol (JUJPAH; Thomas *et al.*, 2015 ▸) adopt the same packing as the phenol mol­ecule in phenol hemihydrate. These are reported in space group *Pnab* rather than the conventional setting of *Pbcn*, otherwise the only meaningful difference is the presence of S—H⋯S or Se—H⋯Se chains long the crystal’s *a* axis instead of rings of O—H⋯O hydrogen bonds *via* H_2_O.

It is worth adding that no matches to the phenol packing motif in Meuthen & von Stackelberg’s (1960 ▸) structure were found in the CSD.

## DFT geometry relaxations   

Zero-pressure athermal geometry optimizations of the phenol hemihydrate structure were performed using Density Functional Theory, DFT, and the plane-wave pseudopotential method (Hohenberg & Kohn, 1964 ▸: Kohn & Sham, 1965 ▸). The calculations were implemented in *CASTEP* v 17.2 (Payne *et al.*, 1992 ▸: Segall *et al.*, 2002 ▸: Clark *et al.*, 2005 ▸) in conjunction with the analysis tools in the *Materials Studio* software package. Ultrasoft pseudopotentials with a basis-set cut-off of 1200 eV and a 2×2×3 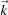-point grid (∼0.04 Å^−1^ reciprocal lattice spacing) were required to achieve convergence of better than 1×10^−2^ GPa in the stress and better than 1×10 ^−3^ eV per atom in total energy. The ‘PBE’ gradient-corrected functional (Perdew *et al.*, 1996 ▸, 1997 ▸) was used in conjunction with both the Grimme (G06) dispersion correction (Grimme, 2006 ▸), the Tkatchenko & Scheffler (TS) dispersion correction (Tkatchenko & Scheffler, 2009 ▸) and the Many-Body Dispersion (MBD) correction (Tkatchenko *et al.*, 2012 ▸).

Structural relaxations were begun from the experimentally determined crystal structure using the BFGS method (Pfrommer *et al.*, 1997 ▸). These were considered to have converged when the forces on each atom were less than 5×10 ^−3^ eV Å^−1^ and each component of the stress tensor was smaller than 0.005 GPa.

Supplementary Tables S4 and S5 report both the inter- and intra­molecular distances and angles found in the PBE + MBD, PBE + TS and PBE + G06 simulations. In each case, the inter­nal geometry of the phenol and the water mol­ecules are nearly identical. However, the inter­molecular contacts differ substanti­ally between the G06 and TS-based (TS & MBD) dispersion corrections, leading to large deviations in the calculated athermal lattice parameters from the observed 10 K unit-cell dimensions. PBE + TS agrees with the experimental values much more closely than PBE + G06, as expected on the basis of a recent computational survey (Binns *et al.*, 2014 ▸). Whilst PBE + MBD gives marginally more accurate inter­molecular distances and matches the 10 K unit-cell volume extremely well, the axial ratios are less accurate than PBE + TS. The structures obtained from the three zero-pressure geometry optimizations are provided in the electronic supplementary information as a CIF.

## Measurement, structure solution and refinement   

**Figure 8:**
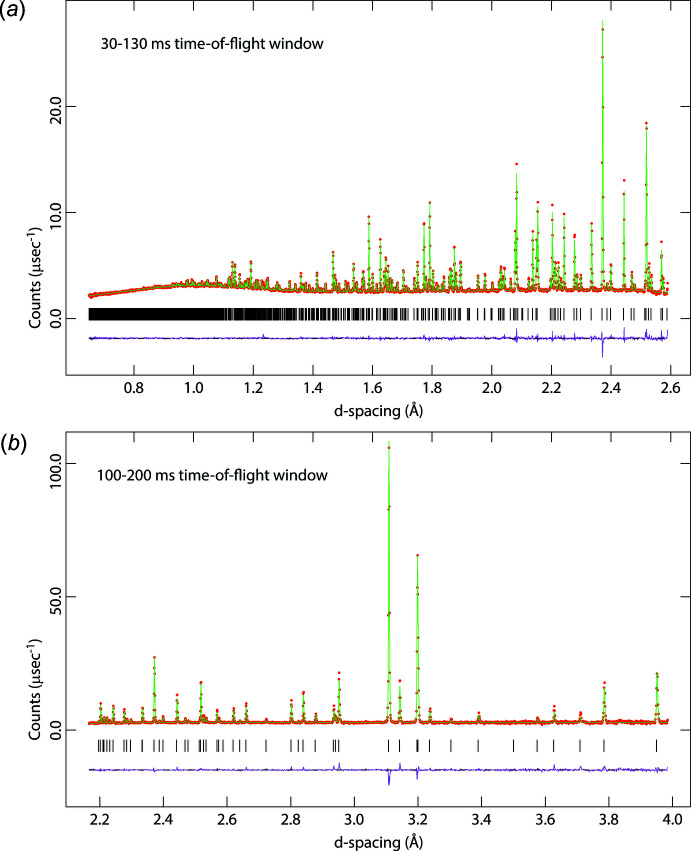
Neutron powder-diffraction data (red circles) measured from phenol hemihydrate at 280 K using HRPD’s 30–130 ms time-of-flight window (*a*) and the 100–200 ms TOF window (*b*). The green line indicates the structural model fit and the purple trace underneath is the difference profile. Black tick marks denote the positions of Bragg peaks.

Crystal data, data collection and structure refinement details are summarized in Table 2 ▸. Neutron powder diffraction data were collected from the sample, mounted in a Closed-Cycle Refrigerator (CCR) on the High Resolution Powder Diffractometer (HRPD) at the ISIS spallation neutron source (Ibberson, 2009 ▸). Initial examination of the specimen at 250 K revealed the presence of ∼3 wt. % D_2_O ice I*h*. An ice-free ‘structural’ dataset with excellent counting statistics was therefore obtained after warming to 280 K. Two 100 ms-wide time-of-flight data-acquisition windows were measured consecutively: 30–130 ms measured for 2 h 25 m (104 µA h); 100–200 ms measured for 56 m (40 µA h). In the instrument’s highest resolution backscattering detector banks (2*θ* = 158–176°) these time windows provide *d*-spacing coverage – after trimming noisier data from the window edges – from 0.65–3.95 Å (Fig. 8 ▸). Data were focussed to a common scattering angle (2*θ* = 168.3°), normalized to the incident spectrum and corrected for instrument efficiency by reference to a V:Nb null-scattering standard using the *Mantid* suite of powder diffraction algorithms (Mantid, 2013 ▸; Arnold *et al.*, 2014 ▸).

Since it was clear that the ‘heavy’ atom structure reported by Meuthen & von Stackelberg (1960 ▸) was not correct, the data were treated *ab initio* as an unknown. The powder data were indexed using *DICVOL06* (Louër & Boultif, 2007 ▸) and examined for systematic absences. This confirmed the crystal system, lattice parameters and space-group assignment of Meuthen & von Stackelberg (1960 ▸), *Pbcn*, to be correct.

Structure solution was done using the parallel tempering algorithm in *FOX*, version 1.9.7.1 (Favre-Nicolin & Černý, 2002 ▸, 2004 ▸), optimizing the position and orientation of a rigid ’ideal’ phenol mol­ecule (C—C = 1.390 Å, C—O = 1.375 Å, C—D = 1.085 Å, and O—D = 0.990 Å; all inter­nal angles of the aromatic ring = 120° and C—O—D = 109°) in order to minimize the difference between the observed and calculated diffraction pattern. In twenty runs of 1/2 million trials each, the minimizations consistently produced identical packing arrangements of the phenol mol­ecules, differing from one another only in the position of the origin. Difference-Fourier maps phased on these structures revealed nuclear scattering density around the 4 *c* sites that corresponded to the bridging water mol­ecule. Subsequent addition of a water mol­ecule to this site and relaxation of the phenol mol­ecule’s inter­nal degrees of freedom provided a solution with the lowest overall cost function, which formed the basis for further analysis.

The trial structure was refined against the 280 K neutron powder dataset using *GSAS*/*Expgui* (Larsen & Von Dreele, 2000 ▸; Toby, 2001 ▸), initially with quite stiff bond-length restraints, and isotropic displacement parameter shifts of similar atoms constrained to be equal. As the refinement progressed, both the bond-length restraints and *U*_iso_ constraints were turned off and all atoms were freely refined with anisotropic displacement parameters. The fit to the data collected at 280 K is shown in Fig. 8 ▸.

**Figure 9:**
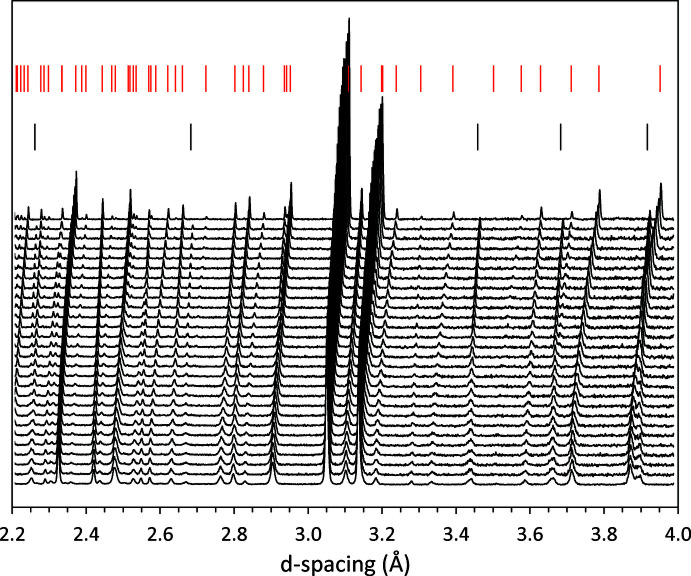
Stack-plot of neutron powder-diffraction data measured on cooling in 10 K increments from 280 K (top) to 10 K (bottom). Red tick marks indicate the Bragg reflections of phenol hemihydrate at 280 K and the black tick marks show the positions of reflections from ice I*h* at 270 K. Note the broadening of all peaks that becomes substantial below ∼140 K.

Additional data were collected in 10 K increments on cooling to 10 K; each datum involved ramping down the set-point at 3 K min^−1^, followed by an equilibration dwell time of 10 m after reaching the set-point, and then a measurement duration of 21 m (15 µA h). These data reveal not only the presence of Bragg peaks from D_2_O ice I*h*, but also the substantial broadening of peaks from phenol hemihydrate. The data shown in Fig. 9 ▸ were used to obtain the lattice parameters given in supplementary Table S1 and analysed in section 5 above.

## Synthesis and crystallization   

Crystalline phenol-*d*_6_ (Sigma Aldrich 176060, 99 atom % D) was mixed with liquid D_2_O (Aldrich 151882, 99.9 atom % D) to form an aqueous solution with a composition equivalent to the stoichiometry of phenol-*d*_6_ hemideuterate (90.910 wt.% phenol-*d*_6_). This liquid was deca­nted dropwise with a glass pipette directly into liquid nitro­gen, forming pinkish-white solid spherules 3–5 mm in diameter. These were transferred into a glass vial and stored in a freezer at ∼255 K for several days. Directly prior to the start of the measurements, the contents of the vial were ground to a fine pale-pink powder under liquid nitro­gen and transferred into a nitro­gen-chilled sample holder. The sample container consisted of a solid aluminium alloy frame with a cuboid central cavity 18 mm x 23 mm (*w* × *h* perpendicular to the incident neutron beam) × 10 mm (depth parallel to the incident beam). The open front and back sides of the sample were covered with vanadium foil windows (125 µm thick), held in place with stainless steel frames and sealed with indium wire. Exposed Al and steel around the ‘front’ vanadium window were masked with Gd and Cd foils. Sample temperatures were monitored with a RhFe thermocouple embedded in the Al frame; active heating was generated by a Watlow Firerod cartridge heater embedded in the opposite side of the sample holder. The whole assembly was mounted on a centre stick and inserted in a closed-cycle refrigerator (CCR) in order to carry out variable-temperature measurements.

## Supplementary Material

Crystal structure: contains datablock(s) PHENOL_HEMI_280K_publ, PHENOL_HEMI_280K_overall, I, PHENOL_HEMI_280K_p_01, PHENOL_HEMI_280K_p_02. DOI: 10.1107/S2056989020007719/wm5564sup1.cif

Electronic supplementary figures and tables. DOI: 10.1107/S2056989020007719/wm5564sup2.pdf

DFT geometry optimizations. DOI: 10.1107/S2056989020007719/wm5564sup3.txt

CCDC reference: 2008230

Additional supporting information:  crystallographic information; 3D view; checkCIF report

## Figures and Tables

**Table 1:** Hydrogen-bond geometry (Å, °) *Cg* is the centroid of the C1–C6 ring located at (0.310, 0.634, 0.451).

*D*—H⋯*A*	*D*—H	H⋯*A*	*D*⋯*A*	*D*—H⋯*A*
O1—D7⋯Ow	0.97 (1)	1.85 (1)	2.793 (7)	165 (1)
Ow—Dw^i^⋯O1^ii^	0.98 (1)	1.83 (1)	2.746 (5)	154 (1)
C1—D1⋯*Cg* ^iii^	1.08 (1)	3.15	3.844	123
C2—D2⋯*Cg* ^iii^	1.06 (1)	3.29	3.897	118
C5—D5⋯*Cg* ^iv^	1.06 (1)	2.89	3.719	136

Symmetry codes: (i) 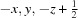; (ii) 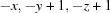; (iii) 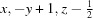; (iv) 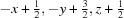.

**Table 2:** Experimental details

Crystal data	
Chemical formula	C_6_D_6_O·0.5D_2_O
*M*_r_	110.16
Crystal system, space group	Orthorhombic, *P* *b* *c* *n*
Temperature (K)	280
*a*, *b*, *c* (Å)	13.21570 (2), 10.89240 (2), 7.902113 (15)
*V* (Å^3^)	1137.52 (1)
*Z*	8
Radiation type	Neutron
Specimen shape, size (mm)	Cuboid, 18 × 23 × 10

Data collection	
Diffractometer	HRPD, High Resolution Neutron Powder Diffractometer
Specimen mounting	Aluminium-framed slab can with vanadium windows, shielded with Gd foil
Data collection mode	Reflection
Scan method	Time of flight
2θ values (°)	2θ_fixed_ = 168.329
Distance from source to specimen (mm)	95000
Distance from specimen to detector (mm)	965

Refinement	
*R* factors and goodness of fit	*R*_p_ = 0.035, *R*_wp_ = 0.019, *R*_exp_ = 0.045, *R*(*F* ^2^) = 0.46850, χ^2^ = 1.904
No. of parameters	153

Computer programs: *HRPD DAE3*, *IBEX* (Akeroyd *et al.*, 2018 ▸), *Mantid* (Arnold *et al.*, 2014 ▸: Mantid, 2013 ▸), *FOX* (Favre-Nicolin & Černý, 2002 ▸, 2004 ▸), *GSAS* (Larsen & Von Dreele, 2000 ▸), *DIAMOND* (Putz & Brandenburg, 2006 ▸), *VESTA* (Momma & Izumi, 2011 ▸) and *publCIF* (Westrip, 2010 ▸).

## supplementary crystallographic information

### Crystal data

**Table:**

C_6_D_6_O·0.5D_2_O	*V* = 1137.52 (1) Å^3^
*M**_r_* = 110.16	*Z* = 8
Orthorhombic, *P**b**c**n*	*F*(000) = 440.0
Hall symbol: -P 2n 2ab	*D*_x_ = 1.286 Mg m^−^^3^
*a* = 13.21570 (2) Å	Melting point: 289 K
*b* = 10.89240 (2) Å	*T* = 280 K
*c* = 7.902113 (15) Å	Particle morphology: fine powder

### Fractional atomic coordinates and isotropic or equivalent isotropic displacement parameters (Å^2^)

**Table:**

	*x*	*y*	*z*	*U*_iso_*/*U*_eq_	
C1	0.2611 (2)	0.5384 (3)	0.3681 (4)	0.06249	
D1	0.2246 (2)	0.4652 (4)	0.2981 (5)	0.09617	
C2	0.3664 (2)	0.5482 (4)	0.3650 (5)	0.07906	
D2	0.4099 (3)	0.4827 (5)	0.2967 (6)	0.12157	
C3	0.4154 (3)	0.6429 (3)	0.4484 (5)	0.07808	
D3	0.4947 (3)	0.6522 (4)	0.4493 (6)	0.11566	
C4	0.3580 (3)	0.7277 (4)	0.5342 (5)	0.07491	
D4	0.3925 (3)	0.8022 (5)	0.6060 (7)	0.13499	
C5	0.2519 (3)	0.7205 (4)	0.5382 (4)	0.06855	
D5	0.2059 (3)	0.7837 (4)	0.6052 (5)	0.10795	
C6	0.2043 (2)	0.6255 (3)	0.4538 (4)	0.06001	
O1	0.1012 (3)	0.6199 (4)	0.4663 (6)	0.07452	
D7	0.0767 (3)	0.5638 (5)	0.3808 (6)	0.08765	
Ow1	0.0	0.4584 (7)	0.25	0.06156	
Dw1	0.0326 (3)	0.4065 (4)	0.1647 (6)	0.09388	

### Atomic displacement parameters (Å^2^)

**Table:**

	*U*^11^	*U*^22^	*U*^33^	*U*^12^	*U*^13^	*U*^23^
C1	0.075 (3)	0.047 (2)	0.066 (3)	−0.0009 (17)	−0.0008 (18)	−0.0091 (17)
D1	0.075 (3)	0.092 (3)	0.121 (4)	−0.004 (2)	0.0041 (18)	−0.049 (3)
C2	0.051 (3)	0.081 (3)	0.105 (4)	0.013 (2)	0.013 (2)	0.001 (2)
D2	0.079 (3)	0.140 (5)	0.145 (4)	0.036 (3)	0.008 (2)	−0.046 (3)
C3	0.052 (2)	0.088 (3)	0.094 (3)	−0.010 (2)	−0.011 (2)	0.001 (2)
D3	0.051 (2)	0.135 (4)	0.161 (4)	−0.003 (2)	−0.005 (3)	−0.005 (3)
C4	0.074 (3)	0.065 (3)	0.086 (3)	−0.012 (2)	0.008 (2)	−0.023 (2)
D4	0.111 (3)	0.096 (4)	0.198 (6)	−0.025 (3)	0.003 (3)	−0.041 (3)
C5	0.063 (3)	0.077 (3)	0.066 (3)	0.0008 (18)	0.0201 (18)	−0.009 (2)
D5	0.076 (2)	0.099 (3)	0.149 (4)	−0.005 (2)	0.021 (3)	−0.059 (2)
C6	0.059 (2)	0.059 (2)	0.0615 (19)	−0.0039 (18)	0.0016 (18)	0.0018 (15)
O1	0.055 (3)	0.085 (4)	0.083 (4)	0.002 (2)	0.009 (2)	−0.024 (3)
D7	0.071 (3)	0.102 (4)	0.090 (3)	0.011 (2)	−0.001 (2)	−0.012 (2)
Ow1	0.067 (4)	0.076 (5)	0.042 (4)	0.0	0.005 (3)	0.0
Dw1	0.078 (2)	0.086 (4)	0.118 (4)	0.009 (2)	0.004 (2)	−0.019 (2)

### Geometric parameters (Å, º)

**Table:**

C1—D1	1.084 (4)	D5—C5	1.060 (4)
C1—C2	1.396 (4)	D5—C6	2.098 (5)
C1—C6	1.387 (4)	C6—C1	1.387 (4)
D1—C1	1.084 (4)	C6—C5	1.382 (4)
C2—C1	1.396 (4)	C6—D5	2.098 (5)
C2—D2	1.063 (4)	C6—O1	1.368 (5)
C2—C3	1.385 (5)	C6—D7	1.905 (5)
D2—C2	1.063 (4)	O1—C6	1.368 (5)
C3—C2	1.385 (5)	O1—D7	0.967 (4)
C3—D3	1.053 (3)	O1—Dw1^i^	1.833 (6)
C3—C4	1.374 (4)	D7—C6	1.905 (5)
D3—C3	1.053 (3)	D7—O1	0.967 (4)
D3—C4	2.096 (5)	D7—Ow1	1.847 (7)
C4—C3	1.374 (4)	Ow1—D7	1.847 (7)
C4—D3	2.096 (5)	Ow1—D7^ii^	1.847 (7)
C4—D4	1.091 (4)	Ow1—Dw1	0.980 (4)
C4—C5	1.405 (4)	Ow1—Dw1^ii^	0.980 (4)
D4—C4	1.091 (4)	Dw1—O1^iii^	1.833 (6)
C5—C4	1.405 (4)	Dw1—Ow1	0.980 (4)
C5—D5	1.060 (4)	Dw1—Dw1^ii^	1.600 (8)
C5—C6	1.382 (4)		
			
D1—C1—C2	119.4 (4)	C3—C4—C5	121.7 (4)
D1—C1—C6	120.8 (4)	D4—C4—C5	116.6 (4)
C2—C1—C6	119.7 (4)	C4—C5—D5	123.2 (4)
C1—C2—D2	119.7 (5)	C4—C5—C6	119.0 (4)
C1—C2—C3	121.0 (4)	D5—C5—C6	117.8 (4)
D2—C2—C3	119.3 (4)	C1—C6—C5	120.1 (3)
C2—C3—D3	122.7 (5)	C1—C6—O1	123.0 (4)
C2—C3—C4	118.5 (4)	C5—C6—O1	116.9 (4)
D3—C3—C4	118.8 (5)	C6—O1—D7	108.2 (5)
C3—C4—D4	121.7 (5)	Dw1—Ow1—Dw1^ii^	109.4 (10)

Symmetry codes: (i) *x*, −*y*+1, *z*+1/2; (ii) −*x*, *y*, −*z*+1/2; (iii) *x*, −*y*+1, *z*−1/2.

### Hydrogen-bond geometry (Å, º)

*Cg* is the centroid of the C1–C6 ring located at (0.310, 0.634, 0.451).

**Table:**

*D*—H···*A*	*D*—H	H···*A*	*D*···*A*	*D*—H···*A*
O1—D7···Ow	0.97 (1)	1.85 (1)	2.793 (7)	165 (1)
Ow—Dw^ii^···O1^iv^	0.98 (1)	1.83 (1)	2.746 (5)	154 (1)
C1—D1···*Cg*^iii^	1.08 (1)	3.15	3.844	123
C2—D2···*Cg*^iii^	1.06 (1)	3.29	3.897	118
C5—D5···*Cg*^v^	1.06 (1)	2.89	3.719	136

Symmetry codes: (ii) −*x*, *y*, −*z*+1/2; (iii) *x*, −*y*+1, *z*−1/2; (iv) −*x*, −*y*+1, −*z*+1; (v) −*x*+1/2, −*y*+3/2, *z*+1/2.

## References

[bb19] Akeroyd, F. A., Baker, K. V. L., Clarke, M. J., Holt, J. R., Howells, G. D., Keymer, D. P., Löhnert, T., Moreton-Smith, C. M., Oram, D. E., Potter, A., Rey, I. H., Willemson, T. A. & Wood, K. (2018). *J. Phys. Conf. Ser.* **1021**, 012019.

[bb1] Alexeev, V. Th. (1883). *Zh. Russ. Fiz. Khim. Obshch*, **15**, 412–413.

[bb2] Allan, D. R., Clark, S. J., Dawson, A., McGregor, P. A. & Parsons, S. (2002). *Acta Cryst.* B**58**, 1018–1024. 10.1107/s0108768102018797 12456981

[bb3] André, D., Fourme, R. & Renaud, M. (1971). *Acta Cryst.* B**27**, 2371–2380.

[bb4] Arnold, O., Bilheux, J. C., Borreguero, J. M., Buts, A., Campbell, S. I., Chapon, L., Doucet, M., Draper, N., Ferraz Leal, R., Gigg, M. A., Lynch, V. E., Markvardsen, A., Mikkelson, D. J., Mikkelson, R. L., Miller, R., Palmen, K., Parker, P., Passos, G., Perring, T. G., Peterson, P. F., Ren, S., Reuter, M. A., Savici, A. T., Taylor, J. W., Taylor, R. J., Tolchenov, R., Zhou, W. & Zikovsky, J. (2014). *Nucl. Instrum. Methods Phys. Res. A*, **764**, 156–166.

[bb5] Binns, J., Healy, M. R., Parsons, S. & Morrison, C. A. (2014). *Acta Cryst.* B**70**, 259–267. 10.1107/S205252061303268X 24675595

[bb6] Biswas, S. G. (1958). *Acta Cryst.* **11**, 882–884.

[bb7] Calvert, F. C. (1865). *J. Chem. Soc.* **18**, 66–70.

[bb8] Chantrapromma, S., Jansrisewangwong, P., Chanawanno, K. & Fun, H.-K. (2011). *Acta Cryst.* E**67**, o2221–o2222. 10.1107/S1600536811029679 PMC3200688 22064463

[bb9] Clark, S. J., Segall, M. D., Pickard, C. J., Hasnip, P. J., Probert, M. I. J., Refson, K. & Payne, M. C. (2005). *Z. Kristallogr.* **220**, 567–570.

[bb10] Demirtaş, G., Dege, N. & Büyükgüngör, O. (2011). *Acta Cryst.* E**67**, o1509–o1510. 10.1107/S1600536811018848 PMC3120380 21754875

[bb11] Favre-Nicolin, V. & Černý, R. (2002). *J. Appl. Cryst.* **35**, 734–743.

[bb12] Favre-Nicolin, V. & Černý, R. (2004). *Z. Kristallogr.* **219**, 847–856.

[bb13] Fortes, A. D. (2019). *Structures of phenol-ammonia and phenol-water compounds*. STFC ISIS Neutron and Muon Source, RB1920009. https://doi.org/10.5286/ISIS.E.RB1920009.

[bb14] Grimme, S. (2006). *J. Comput. Chem.* **27**, 1787–1799. 10.1002/jcc.20495 16955487

[bb15] Groom, C. R., Bruno, I. J., Lightfoot, M. P. & Ward, S. C. (2016). *Acta Cryst.* B**72**, 171–179. 10.1107/S2052520616003954 PMC4822653 27048719

[bb16] Hohenberg, P. & Kohn, W. (1964). *Phys. Rev.* **136**, B864–B871.

[bb17] Ibberson, R. M. (2009). *Nucl. Instrum. Methods Phys. Res. A*, **600**, 47–49.

[bb18] Kohn, W. & Sham, L. J. (1965). *Phys. Rev.* **140**, A1133–A1138.

[bb20] Larsen, A. C. & Von Dreele, R. B. (2000). *General Structure Analysis System* (*GSAS*). Los Alamos National Laboratory Report LAUR 86-748, Los Alamos, New Mexico, USA.

[bb21] Li, H.-P., Yang, Y.-X. & Ng, S. W. (2010). *Acta Cryst.* E**66**, o2867. 10.1107/S1600536810041425 PMC3009157 21589049

[bb22] Louër, D. & Boultif, A. (2007). *Z. Kristallogr. Suppl.* pp. 191–196.

[bb23] Mantid (2013). *Manipulation and Analysis Toolkit for Instrument Data; Mantid Project.* http://dx.doi.org/10.5286/SOFTWARE/MANTID.

[bb24] McKinnon, J. J., Jayatilaka, D. & Spackman, M. A. (2007). *Chem. Commun.* pp. 3814–3816. 10.1039/b704980c 18217656

[bb25] Merz, K. (2006). *Cryst. Growth Des.* **6**, 1615–1619.

[bb26] Meuthen, B. & von Stackelberg, M. (1960). *Z. Elektrochem*, **64**, 387–390.

[bb27] Momma, K. & Izumi, F. (2011). *J. Appl. Cryst.* **44**, 1272–1276.

[bb28] Nath, N. K. & Naumov, P. (2015). *Maced. J. Chem. Chem. Eng.* **34**, 63–66.

[bb29] Pachler, K. & Von Stackelberg, M. (1963). *Z. Kristallogr.* **119**, 15–29.

[bb30] Paternò, E. & Ampola, G. (1897). *Gazz. Chim. Ital.* **27**, 481–536.

[bb31] Payne, M. C., Teter, M. P., Allan, D. C., Arias, T. A. & Joannopoulos, J. D. (1992). *Rev. Mod. Phys.* **64**, 1045–1097.

[bb32] Perdew, J. P., Burke, K. & Ernzerhof, M. (1996). *Phys. Rev. Lett.* **77**, 3865–3868. 10.1103/PhysRevLett.77.3865 10062328

[bb33] Perdew, J. P., Burke, K. & Ernzerhof, M. (1997). *Phys. Rev. Lett.* **78**, 1396. 10.1103/PhysRevLett.77.3865 10062328

[bb34] Pfrommer, B. G., Côté, M., Louie, S. G. & Cohen, M. L. (1997). *J. Comput. Phys.* **131**, 233–240.

[bb35] Putz, H. & Brandenburg, K. (2006). *Diamond - Crystal and Molecular Structure Visualization*. Crystal Impact - GbR, Kreuzherrenstr. 102, 53227 Bonn, Germany. (http://www.crystalimpact.com/diamond)

[bb36] Rhodes, F. H. & Markley, A. L. (1921). *J. Phys. Chem.* **25**, 527–534.

[bb37] Segall, M. D., Lindan, P. J. D., Probert, M. J., Pickard, C. J., Hasnip, P. J., Clark, S. J. & Payne, M. C. (2002). *J. Phys. Condens. Matter*, **14**, 2717–2744.

[bb38] Smith, R. A. (1932). *Mikrochemie*, **11**, 227–236.

[bb39] Smits, A. & Maarse, J. (1911). *Kong. Nederl. Akad. Wetensch*, **14**, 192–195.

[bb40] Spackman, M. A. & Jayatilaka, D. (2009). *CrystEngComm*, **11**, 19–32.

[bb41] Thomas, S. P., Sathishkumar, R. & Guru Row, T. N. (2015). *Chem. Commun.* **51**, 14255–14258. 10.1039/c5cc03322e 26263890

[bb42] Tkatchenko, A., DiStasio, R. A., Car, R. & Scheffler, M. (2012). *Phys. Rev. Lett.* **108**, 236402. 10.1103/PhysRevLett.108.236402 23003978

[bb43] Tkatchenko, A. & Scheffler, M. (2009). *Phys. Rev. Lett.* **102**, 073005–073008. 10.1103/PhysRevLett.102.073005 19257665

[bb44] Toby, B. H. (2001). *J. Appl. Cryst.* **34**, 210–213.

[bb45] Turner, M. J., McKinnon, J. J., Wolff, S. K., Grimwood, D. J., Spackman, P. R., Jayatilaka, D. & Spackman, M. A. (2017). *CrystalExplorer17*. University of Western Australia. (http://hirshfeldsurface. net)

[bb46] Westrip, S. P. (2010). *J. Appl. Cryst.* **43**, 920–925.

[bb47] Zavodnik, V. E., Bel’skii, V. K. & Zorkii, P. M. (1988). *J. Struct. Chem.* **28**, 793–795.

[bb48] Zhang, X. Q., Mou, X. F., Mao, N., Hao, J. J., Liu, M., Zheng, J. Y., Wang, C. Y., Gu, Y. C. & Shao, C. L. (2018). *Eur. J. Med. Chem.* **146**, 232–244. 10.1016/j.ejmech.2018.01.057 29407953

