# Supplementary material for: Phenol hemihydrate: redetermination of the crystal structure by neutron powder diffraction, Hirshfeld surface analysis and characterization of the thermal expansion
Source: Acta Crystallogr E Crystallogr Commun. 2020 Jun 12;76(Pt 7):1062–9. doi: 10.1107/S2056989020007719 (PMC7336780; doi:10.1107/S2056989020007719)
Supplement: Supplementary file 2 [file e-76-01062-sup2.pdf]

Phenol hemihydrate: redetermination of the crystal structure, Hirschfeld surface analysis and characterisation of the thermal expansion.

A. Dominic Fortes[1,*]

[1]ISIS Pulsed Neutron and Muon Source, Rutherford Appleton Laboratory, Harwell Science and Innovation Campus, Chilton, Oxfordshire OX11 0QX

*Corresponding authors email: dominic.fortes@stfc.ac.uk

SUPPLEMENTARY FIGURES

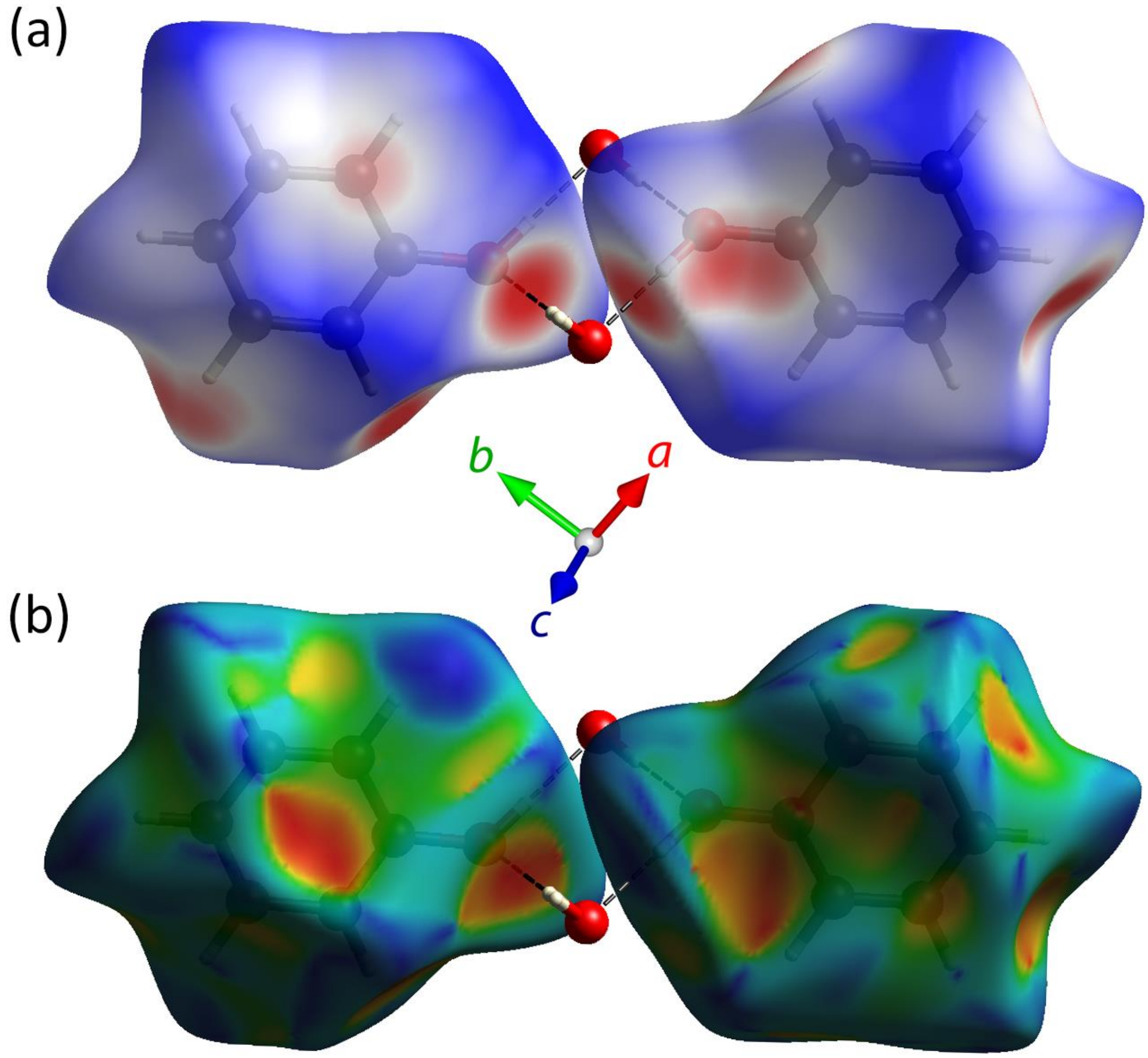

Figure S1

Hirschfeld surface of the phenol molecules in Meuthen & Stackelberg's structure [Meuthen, B. & von Stackelberg, M. (1959). *Z. Elektrochem*. **64**, 387–390.], coloured according to d_{norm} values (a) and using the Shape Index (b). As described in the main text (Figure 1 caption), the structure was completed by addition of hydrogen atoms and calculation of the Hirschfeld surface with CrystalExplorer 17.5

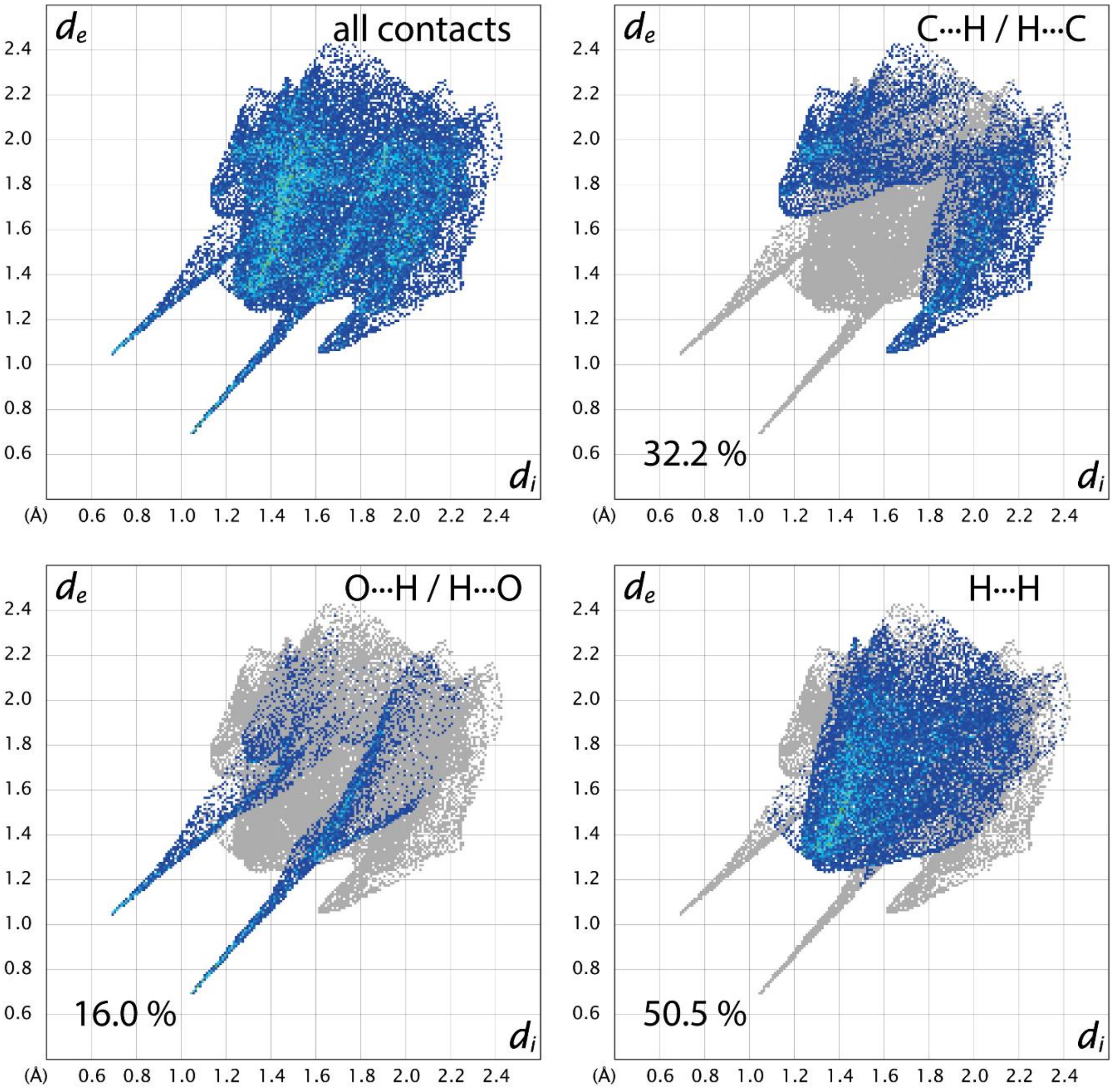

Figure S2

Fingerprint plots for solid phenol.

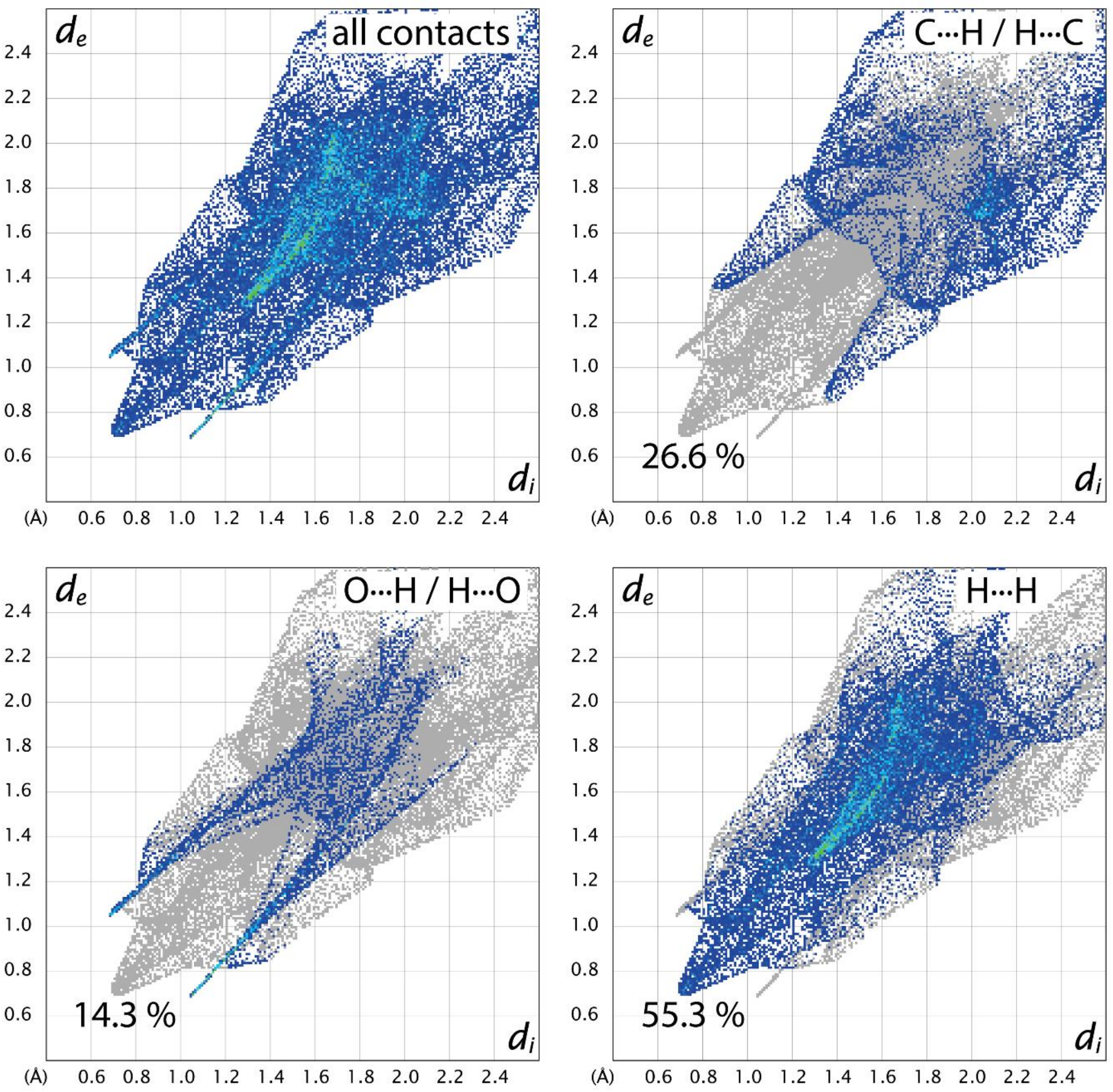

Figure S3

Fingerprint plots for Meuthen & Stackelberg's structure of phenol hemihydrate.

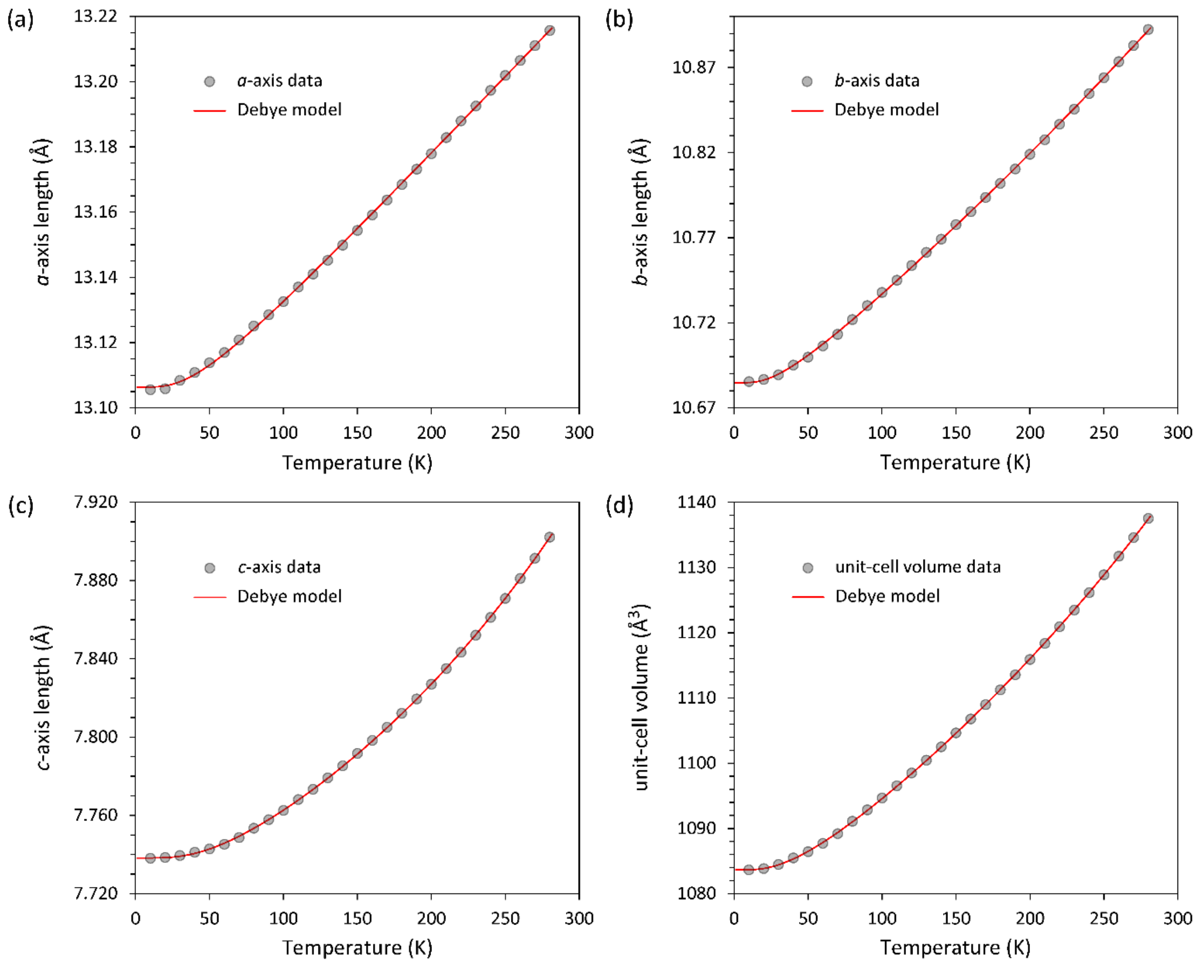

Figure S4

Unit-cell parameters of perdeuterated phenol hemihydrate between 10 and 280 K using the data provided in Supplementary Table S1. Solid lines represent the 2nd order Debye model fit (see main text and Suppl. Table S2). Error bars are smaller than the symbols.

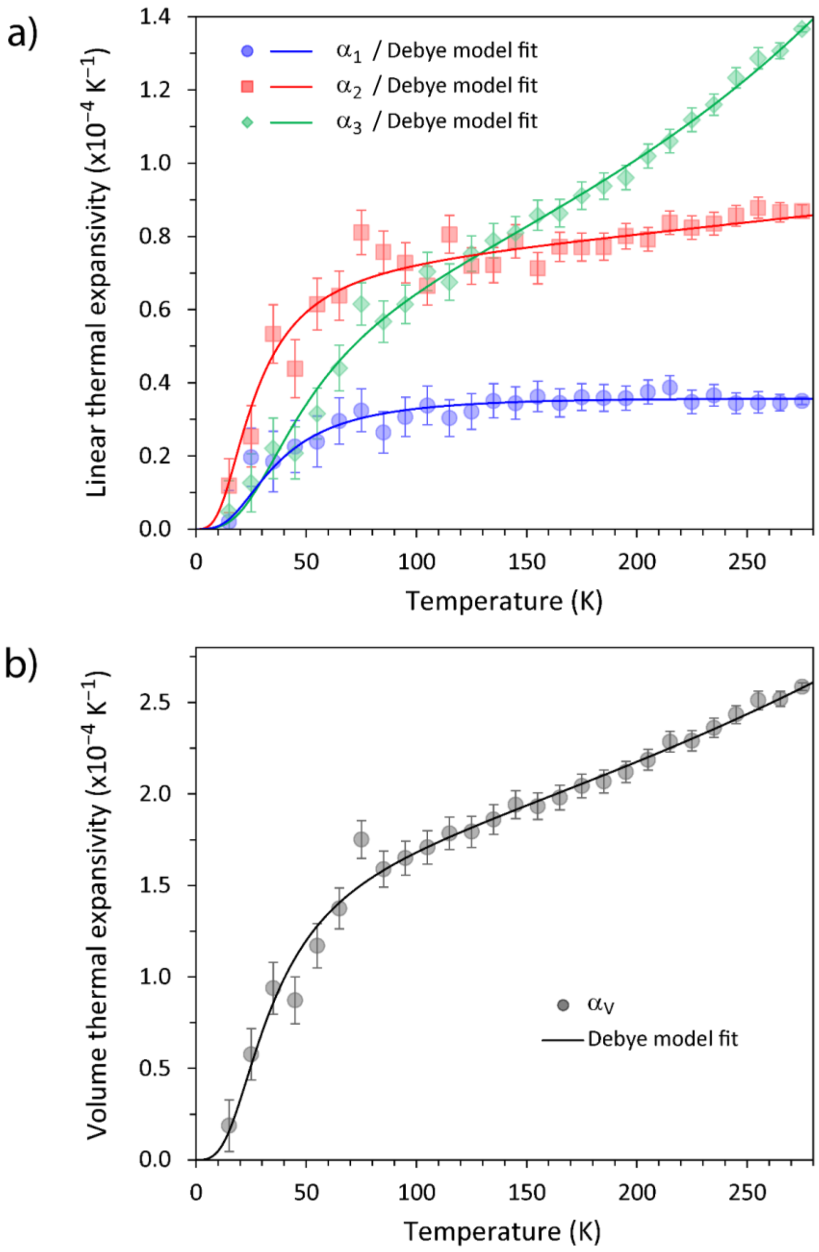

Figure S5

(a) Coefficients of linear thermal expansion and (b) volume thermal expansion for perdeuterated phenol hemihydrate. Symbols are simple point-by-point derivatives of the unit-cell parameters, and the solid lines are derived from the Debye model fit.

SUPPLEMENTARY TABLES

Table S1

Refined unit-cell parameters of perdeuterated phenol hemihydrate measured on cooling from 280 to 10 K. These data are plotted in Supplementary Figure S4.

T (K)	*a* (Å)	*b* (Å)	*c* (Å)	V (Å^3)
280	13.21570(2)	10.89240(2)	7.90211(2)	1137.515(3)
270	13.21107(10)	10.88293(7)	7.89132(4)	1134.58(1)
260	13.20650(13)	10.87351(10)	7.88100(5)	1131.72(1)
250	13.20193(12)	10.86396(10)	7.87086(5)	1128.88(1)
240	13.19739(13)	10.85466(10)	7.86115(5)	1126.13(1)
230	13.19256(14)	10.84559(11)	7.85203(5)	1123.48(1)
220	13.18797(14)	10.83666(11)	7.84324(5)	1120.91(1)
210	13.18286(14)	10.82758(11)	7.83492(5)	1118.35(1)
200	13.17792(14)	10.81901(11)	7.82694(5)	1115.90(1)
190	13.17321(16)	10.81034(12)	7.81942(6)	1113.54(1)
180	13.16848(16)	10.80200(13)	7.81209(6)	1111.24(2)
170	13.16372(14)	10.79368(14)	7.80497(6)	1108.97(1)
160	13.15918(16)	10.78535(16)	7.79823(7)	1106.77(2)
150	13.15441(17)	10.77766(17)	7.79155(7)	1104.64(2)
140	13.14988(17)	10.76918(18)	7.78523(8)	1102.49(2)
130	13.14527(18)	10.76140(19)	7.77910(8)	1100.44(2)
120	13.14104(19)	10.75367(19)	7.77324(8)	1098.47(2)
110	13.13705(20)	10.74500(21)	7.76799(8)	1096.51(2)
100	13.13261(20)	10.73786(20)	7.76252(8)	1094.64(2)
90	13.12858(22)	10.73004(22)	7.75775(9)	1092.84(2)
80	13.12511(22)	10.72192(22)	7.75335(9)	1091.10(2)
70	13.12085(24)	10.71322(24)	7.74858(9)	1089.19(2)
60	13.11696(26)	10.70639(27)	7.74517(10)	1087.70(3)
50	13.11382(26)	10.69980(28)	7.74273(11)	1086.42(3)
40	13.11086(27)	10.69511(35)	7.74111(11)	1085.48(3)
30	13.10844(28)	10.68940(32)	7.73941(12)	1084.46(3)
20	13.10586(31)	10.68669(34)	7.73842(13)	1083.83(3)
10	13.10559(24)	10.68542(26)	7.73806(10)	1083.63(2)

Table S2

Parameters obtained from fitting of a 2nd order Debye model to the unit-cell parameters given in Table S2. The fits are plotted in Suppl. Fig. S4.

	a^3	b^3	c^3	V
θ_D (K)	152(4)	108(6)	209(5)	138(3)
X_0 (cm^3 mol^{-1})	338.95(2)	183.65(3)	69.761(4)	163.144(9)
Q (x10^4 J cm^{-3})	651(4)	330(6)	345(5)	436(4)
b	0	2.0(2)	7.4(2)	5.9(2)
		Derived parameters		
X_0 (Å, Å^3)	13.106(2)	10.685(3)	7.738(1)	1083.63(11)
K_0/γ	19.2(1)	18.0(3)	49.5(7)	26.7(3)
K_0'	1	5.0(5)	15.8(4)	12.8(4)

Table S3

Comparison between the experimentally-determined unit-cell parameters of phenol hemihydrate at 10 K and those from Density Functional Theory (DFT) calculations using several different van der Waals corrections (see main text). The relative difference with the experimental values are reported in red.

	Experimental	Athermal DFT					
	10 K	PBE + MBD		PBE + TS		PBE + G06	
a (Å)	13.1056(2)	13.0747	(−0.24 %)	13.1334	(+0.21 %)	12.9747	(−1.00 %)
b (Å)	10.6854(3)	10.9656	(+2.62 %)	10.6781	(−0.07 %)	10.5449	(−1.32 %)
c (Å)	7.7381(1)	7.5822	(−2.01 %)	7.6125	(−1.62 %)	7.4800	(−3.33 %)
V ($Å^3$)	1083.63(2)	1087.0747	(+0.32 %)	1067.5752	(−1.48 %)	1023.3905	(−5.56 %)
b/*a*	0.81533(3)	0.8387	(+2.87 %)	0.8130	(−0.23 %)	0.8127	(−0.32 %)
c/*a*	0.59044(1)	0.5799	(−1.79 %)	0.5796	(−1.84 %)	0.5765	(−2.36 %)

Table S4

Comparison between the intramolecular geometry found experimentally at 280 K in phenol hemihydrate with values obtained from DFT calculations using several different van der Waals corrections (see main text). All distances are in Å units and angles in degrees.

	Experimental 280 K	Athermal DFT		
		PBE + MBD	PBE + TS	PBE + G06
C1–C2	1.3956(45)	1.39288	1.39228	1.39367
C2–C3	1.3849(55)	1.39387	1.39390	1.39518
C3–C4	1.3747(56)	1.39440	1.39445	1.39584
C4–C5	1.4047(54)	1.39209	1.39136	1.39263
C5–C6	1.3825(51)	1.39404	1.39331	1.39422
C6–C1	1.3864(45)	1.39630	1.39528	1.39601
C1–D1	1.0837(52)	1.08910	1.08766	1.08870
C2–D2	1.0631(63)	1.08989	1.08879	1.08958
C3–D3	1.0531(51)	1.08912	1.08807	1.08872
C4–D4	1.0902(67)	1.08944	1.08830	1.08910
C5–D5	1.0600(56)	1.08959	1.08812	1.08913
C6–O1	1.3679(51)	1.37834	1.37795	1.37823
O1–D7	0.9667(67)	1.00958	1.00776	1.01007
C6–O1–D7	108.13(44)	111.2367	111.2514	111.1080
C1–C6–O1–D7	17.08(63)	14.1947	14.0484	10.9798
Ow–Dw	0.9797(62)	1.00167	0.99954	1.00156
Dw–Ow–Dw$^{(i)}$	109.52(54)	111.4704	111.3403	111.7708

Symmetry codes:

(i) −*x*, *y*, ½−*z*

Table S5

Comparison between the intermolecular geometry found experimentally at 280 K in phenol hemihydrate with values obtained from DFT calculations using several different van der Waals corrections (see main text). All distances are in Å units and angles in degrees.

	Experimental 280 K	Athermal DFT		
		PBE + MBD	PBE + TS	PBE + G06
O1···Ow	2.7934(66)	2.66578	2.67264	2.64854
D7···Ow	1.8474(67)	1.66912	1.67882	1.63152
O1–D7···Ow	165.32(51)	168.3822	167.9337	168.3014
Ow···O1$^{(ii)}$	2.7459(52)	2.67635	2.67587	2.65755
Dw$^{(i)}$ ···O1$^{(ii)}$	1.8332(64)	1.72287	1.73303	1.70796
Ow–Dw$^{(i)}$ ···O1$^{(ii)}$	153.68(47)	157.6071	155.7102	156.7016
Cg···*Cg*$^{(iii)}$	4.911	4.783	4.754	4.684
Cg···*Cg*$^{(iv)}$	4.948	4.837	4.810	4.748
Cg$^{(iii)}$···*Cg*···*Cg*$^{(iv)}$	70.20	72.68	71.14	71.46
C1···*Cg*$^{(iii)}$	3.844	3.717	3.701	3.621
H1···*Cg*$^{(iii)}$	3.152	3.079	3.079	2.995
C1–H1···*Cg*$^{(iii)}$	122.52	118.01	116.92	116.93
C2···*Cg*$^{(iii)}$	3.897	3.752	3.734	3.650
H2···*Cg*$^{(iii)}$	3.290	3.129	3.127	3.032
C2–H2···*Cg*$^{(iii)}$	117.55	116.99	115.95	116.46
C5···*Cg*$^{(iv)}$	3.719	3.589	3.575	3.497
H5···*Cg*$^{(iv)}$	2.886	2.726	2.734	2.634
C5–H5···*Cg*$^{(iv)}$	135.71	135.85	133.84	135.60

Symmetry codes:

(i) −*x*, *y*, ½−z; (ii) −*x*, 1−*y*, 1−z; (iii) *x*, 1−*y*, −½+z; (iv) ½−*x*, 1½−*y*, ½+*z*
